# To respond or not to respond, the recurring question in plant mechanosensitivity

**DOI:** 10.3389/fpls.2014.00401

**Published:** 2014-08-14

**Authors:** Nathalie Leblanc-Fournier, Ludovic Martin, Catherine Lenne, Mélanie Decourteix

**Affiliations:** ^1^Clermont Université – Université Blaise Pascal, UMR547 PIAF, Clermont-FerrandFrance; ^2^INRA, UMR547 PIAF, Clermont-FerrandFrance; ^3^Laboratoire de Biologie du Développement des Plantes, UMR 7265, Centre National de la Recherche Scientifique/Commissariat à l’Energie Atomique/Aix-Marseille Université, Saint-Paul-lez-DuranceFrance

**Keywords:** mechanosensitivity, accommodation, mechanical stimulus, abiotic stress, wind, acclimation to stress

## Abstract

In nature, terrestrial plants experience many kinds of external mechanical stimulation and respond by triggering a network of signaling events to acclimate their growth and development. Some environmental cues, especially wind, recur on time scales varying from seconds to days. Plants thus have to adapt their sensitivity to such stimulations to avoid constitutive activation of stress responses. The study of plant mechanosensing has been attracting more interest in the last two decades, but plant responses to repetitive mechanical stimulation have yet to be described in detail. In this mini review, alongside classic experiments we survey recent descriptions of the kinetics of plant responses to recurrent stimulation. The ability of plants to modulate their responses to recurrent stimulation at the molecular, cellular, or organ scale is also relevant to other abiotic stimuli. It is possible that plants reduce their responsiveness to environmental signals as a function of their recurrence, recovering full sensitivity several days later. Finally, putative mechanisms underlying mechanosensing regulation are discussed.

## INTRODUCTION

Mechanosensing is an important factor regulating plant growth and development (Hamant, 2013). Mechanical cues may be internal signals produced during tissue or cellular expansion (Ingber, 2005; Hamant et al., 2008) or external signals from the environment, mainly from wind (Moulia et al., 2011). To understand the influence of wind on plant development, different methods have been used in the laboratory to simulate the mechanical effect of wind, like bending, touching, shaking, or brushing the aboveground parts of plants. These mechanical stimulations result in a thigmomorphogenetic syndrome generally characterized by reduction in stem height, modification of the mechanical properties of the stem, increase in root biomass and local increases in stem radial growth depending on the species (Telewski, 2006).

These physiological responses that alter the growth trajectory and form of the plants are thought to be involved in a long-term process of acclimation, tending to reduce the impact of subsequent mechanical stimulation (Moulia et al., 2006; Telewski, 2006). However, less is known about how plants respond to rapid recurrent mechanical stimulations. Wind typically induces repeated flexing of plant organs at different frequencies (De Langre, 2008). Plant stems may oscillate at frequencies in the range of 1–5 Hz in wind, corresponding to 60–300 bends per minute (Rodriguez et al., 2008; Der Loughian et al., 2014). In temperate climates, windy and calm days alternate on a time scale of several days (Stull, 1988). If every response to each mechanical stimulus was of the same magnitude, plants would invest greatly in withstanding mechanical perturbation to the detriment of growth. Plants thus need to permanently fine-tune their response to mechanical stimulation in order to avoid the cost of a constitutive protection system. This holds especially true for trees because of their long-term growth period and their high stature. There is some experimental evidence that plants reduce their responsiveness to mechanical signals as a function of their mechanical history. This acclimation of mechanosensitivity has been named accommodation (Moulia et al., 2011) in reference to the cellular accommodation that bones undergo in response to external mechanical loading (Schriefer et al., 2005). Here we summarize and discuss experimental observations of plants responding to recurrent mechanical simulation. Regulation of plant responsiveness to recurrent cold and drought stress is compared to mechanosensitivity accommodation. Finally, preliminary evidence and speculation about the molecular mechanisms involved in such processes are discussed.

## SATURATION AND DESENSITIZATION, WAYS TO DEAL WITH SUCCESSIVE MECHANICAL STIMULATION

When studying variation in plant responsiveness to successive mechanical stimulations, two parameters need to be taken into account. (i) The intensity of the mechanical stimulation should be quantified so it can be reproducibly and repeatedly applied. (ii) The kinetics of plant responses to single and successive stimulations should be characterized.

**FIGURE 1:**
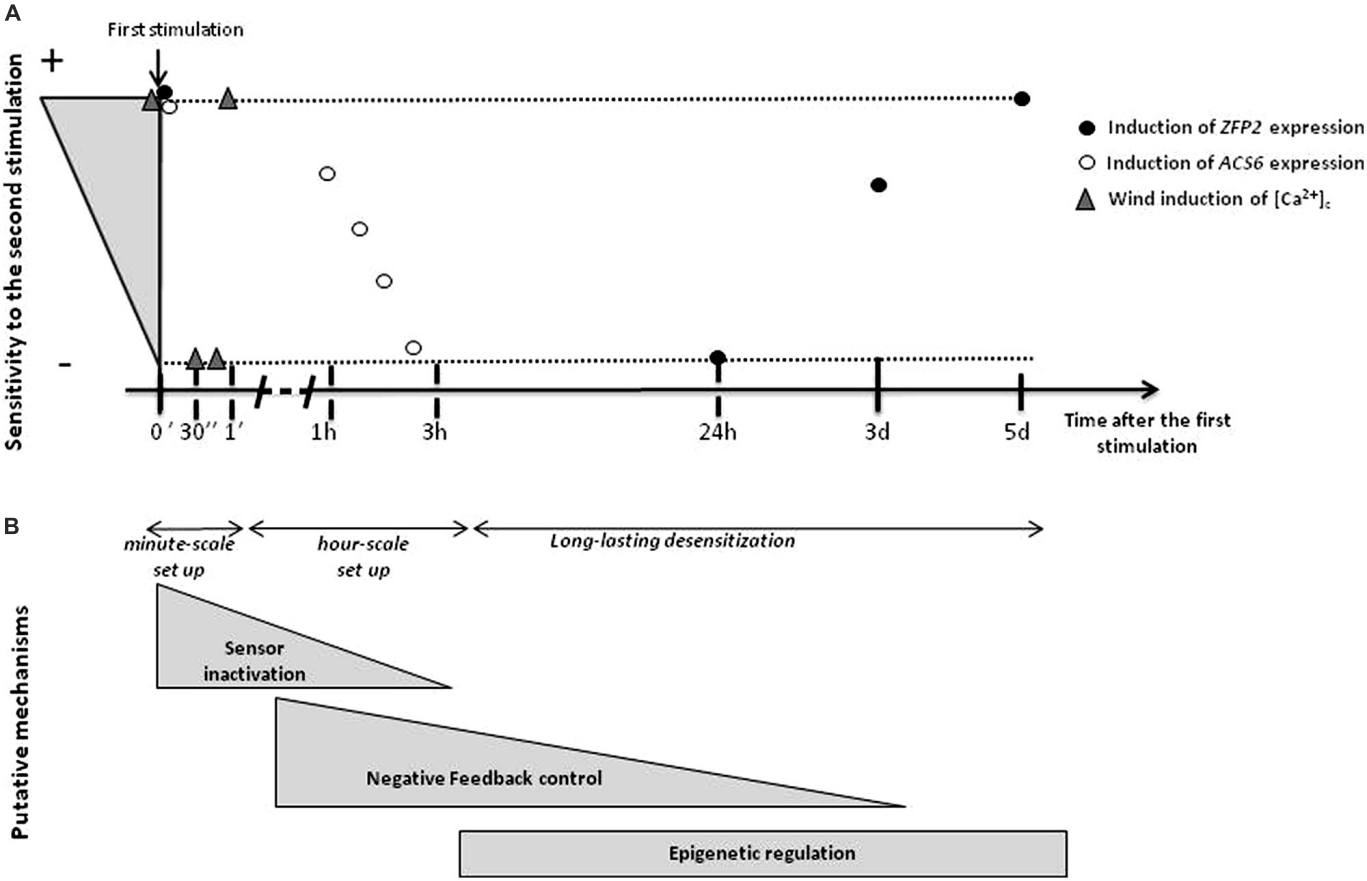
**(A)** Desensitization kinetics set up in response to recurrent mechanical stimulations applied at high frequency in case of wind induced Ca^2+^cytosolic concentration in *Nicotiana tabacum* (Knight et al., 1992) or at low frequency in case of touch-induced *ACS6* expression in *Arabidopsis* (Arteca and Arteca, 1999) and bending-induced *ZFP2* expression in *Populus tremula x P. alba* (Martin et al., 2010). **(B)** Hypothetical mechanisms for the short-term or long-lasting desensitization: (i) alterations of perception through sensor turnover, modification or in activation, (ii) negative feedback from long-term accumulation of signaling molecules or transcription factors and (iii) information storage through epigenetic regulation.

Formerly, a first approach to investigate plant mechanosensitivity was to quantify and compare the effect of different magnitudes or numbers of mechanical stimulations. When *Phaseolus vulgaris* internodes are rubbed repeatedly (following a standardized method), the amount of mechanical stimulation correlates positively with the extent of internode elongation, but the sensory function becomes saturated even with small amounts of rubbing (Jaffe et al., 1980). To mimic the effect of wind, mechanical experiments on tree species are usually done by bending the stem for a few seconds then releasing it. This transitory stimulus (which will be here called bending) has the added advantage of allowing the experimenter to control how much strain is applied, so the physical stimulus perceived by plant cells is known (Coutand and Moulia, 2000; Coutand et al., 2009). In such experiments on *Ulmus americana*, no increment in the secondary growth response was detected after three weeks when stimulation frequency was increased from 5–80 bends a day (Telewski and Pruyn, 1998). In another set of observations on *Prunus persica,* stems were bent in a controlled manner eight times a day. Over the 6 weeks of the experiment, the stimulus of repeated bending affected growth less, even when the actual strains applied were increased slightly over time to compensate for stem radial growth (Coutand et al., 2008). Altogether, these results suggest that saturation of either the mechanosensory or the response systems was reached. However, because the responses were measured at the end of several weeks of treatment, it was not possible to exclude the possibility that plant sensitivity was adjusted with each successive bend and that only some of the first mechanical treatments were responsible for the observed responses. In poplar, controlled stem flexing at a sub-saturation level was coupled with kinetics analyses of the responses to each successive bending. The result was a rapid reduction in responsiveness of both radial growth and gene expression (Martin et al., 2010). In particular, these experiments showed that the second bending, 24 h after the first, was markedly weaker in inducing four early mechanoresponsive genes encoding, respectively, two calmodulins, a C2H2 transcription factor (**Figure 1A**) and a xyloglucan endotransglycosylase. As the abundance of these genes transcripts returned to basal levels before 24 h (Martin et al., 2009), the expression levels observed could only have been due to the second bending and not due to response saturation. These data demonstrated that a single bending is sufficient to initiate a change in plant responsiveness, in this case a down-regulation or desensitization, to subsequent bending. In *Arabidopsis*, an even faster desensitization occurred after unquantified successive touch stimulation (Arteca and Arteca, 1999). As soon as 1 h after the first stimulation, a second touch was less effective in inducing *ACS6* expression, a gene encoding 1-aminocyclopropane-1-carboxylic acid (ACC) synthase enzyme (**Figure 1A**). In the conditions described above after the second bending of the poplar stem, about seven days without any bending stimulus were necessary to recover the full capacity for induction of gene expression (**Figure 1A**, Martin et al., 2010), suggesting that this state of desensitization to mechanical loads lasts for several days. Such desenzitisation was also observed in case of the typical defensive leaf-folding reflex of Mimosa plants (Gagliano et al., 2014). However, in this case, the resenzitisation was assessed with a mechanical disturbance different from the one responsible of the desenzitisation.

The above kinetics could be said to more closely mimic the alternation between windy and calm days in nature rather than oscillations in the wind at frequencies between 1 and 5 Hz (Rodriguez et al., 2008). The effects of mechanical treatments that recur at short intervals were investigated by analyzing rapid cellular events. Knight et al. (1992) showed that wind-induced mechanical stimulations of *Nicotiana* seedlings generate peaks of elevated cytosolic calcium concentrations. The amplitudes of calcium peaks diminished when stimulations were repeated every 5 s until cells became refractory to further stimulation. Full desensitization was attained after about 6–7 stimulations (about 30 s of intermittent stimulation) and full responsiveness was recovered less than 60 s later when stimulation was decreased (**Figure 1A**). However, attenuation of this type of calcium response was not detected in *Arabidopsis* roots when the two touch stimuli were separated by 20 s (Monshausen et al., 2009).

Intuitively we would expect down-regulation of sensitivity upon prolonged mechanical or recurrent stimulation to be a key component of any biological sensory process. However, kinetics and mechanistic data are still lacking to fully describe desensitization. Moreover, the link between the decrease in responsiveness of mechanoresponsive genes and the regulation of mechanoperception is yet to be elucidated.

## FINE-TUNING SENSITIVITY, A RECURRING THEME

Modifying plant responsiveness is not restricted to recurrent mechanical stimuli as other abiotic stimuli act in a similar way. However, a plethora of terminologies have been used to describe these different stress situations which may mask some of the similarities. Generally, altered physiological responses to recurrent abiotic stress that allow a plant to maintain its performances despite the stress, is referred to as “acclimation” or “hardening.” For biotic stress, the term “priming” is usually preferred, and is defined as “the phenomenon whereby previous exposure makes a plant more resistant to future exposure” (Bruce et al., 2007). When the response to a stimulus is modified at the cellular or molecular level, authors predominantly use terms to describe the state of sensitivity of the cells. For example, to describe how peaks of cytosolic free calcium concentration decrease with repeated stimulation/stress, the terms “attenuation” or “desensitization” are used (Knight et al., 1992; Plieth et al., 1999). Bruce et al. (2007) suggested the general term of “stress imprint” to designate “a genetic or biochemical modification of a plant that occurs after stress exposure that causes future responses to future stresses to be different.” This is less anthropomorphic than the concept of “memory” or “training,” but many authors are using “plant stress memory” to encapsulate the idea that plants store information related to a first stress exposure, leading to increased or decreased responses to subsequent exposures (Galis et al., 2009; Stork et al., 2009; Ding et al., 2013).

The time-courses of desensitization–resensitization phenomena have barely been investigated. The kinetics of the generation of peaks in intracellular calcium concentration in response to cold were studied in *Arabidopsis* roots with regimes of recurrent cold stimulation at a range of intervals on the time-scale of several minutes. As in wind-stimulated *Nicotiana* seedlings (Knight et al., 1992), attenuation of the height of calcium peaks was observed and desensitization started minutes after the first stimulation and lasted for a minimum of 30 min before re-sensitization occurred (Plieth et al., 1999). However, this desensitization was overridden if the intensity of the subsequent stimulus was increased (i.e., with a decrease in the temperature). These phases of desensitization or attenuation at the calcium level could avoid an over-mobilization of internal and external stores of calcium which would hamper any further response.

Exposing *Arabidopsis* plants to cold temperatures (+4°C) also triggers rapid modifications in gene expression. One example is the up-regulation of genes encoding members of the AP2/EREBP family of DNA-binding proteins, the cold binding factors (CBFs; Gilmour et al., 1998). Using the accumulation of CBF transcripts as a marker, Zarka et al. (2003) found that the cold-sensing mechanism can be desensitized within a few hours of exposure to a low temperature. In the case of desensitization to +4°C, resensitization (i.e., recovery of *CBF* induction at this same temperature), took between 8 and 24 h of re-exposure to warm temperatures. As for the calcium response, the desensitized state could be overridden by a further decrease in temperature.

In the phenomena described above, desensitization is a way for plants to avoid over-responding, using excess signaling molecules (e.g., calcium) or saturating the sensing machinery. However, in the case of plant defense (Galis et al., 2009; Conrath, 2011) or dehydration (Ding et al., 2012), adjustments in plant sensitivity can also make plants more responsive to subsequent exposures, in what we could call hypersensitization. For example, *Arabidopsis* plants trained by a first exposure to a dehydration stress wilt more slowly than untrained plants when they are exposed a second time. When the plants are subjected to cycles of 2 h of dehydration and 22 h of rehydration, “trainable” genes (e.g., *RD29B*, *RAB18*) produced higher transcript levels in response to subsequent stresses than to the initial stress, whereas “non-trainable” genes expressed similar transcript levels for each stress events (e.g., *RD29A* and *COR15A*). This “transcriptional memory” persisted for at least 5 days and was lost after 7 days (Ding et al., 2012). More recently, a large-scale transcriptional analysis revealed there are 1963 such “memory” genes in the *Arabidopsis* transcriptional network triggered by dehydration stress (Ding et al., 2013).

To conclude, different plant species modify their sensitivity to abiotic stresses at the physiological, cellular and molecular levels with very different kinetics. However, it should be noted that the different mechanisms have some features in common despite the very different environmental cues. For example, at the level of the molecular response, desensitization is usually rapid and lasts for several days.

## HOW TO CONTROL DESENSITIZATION

If desensitization occurs in response to a variety of recurring biotic and abiotic cues, how have the common features of responsiveness regulation emerged in these different signaling pathways? What are the underlying mechanisms? Three potential mechanisms could be proposed depending on the kinetics of their occurrence (**Figure 1B**).

### ALTERATIONS OF PERCEPTION THROUGH SENSOR TURNOVER, MODIFICATION OR INACTIVATION

The natures of potentially numerous touch, cold, and osmotic sensors are still to be fully elucidated. As discussed by Telewski (2006), abiotic stimuli cause membrane deformation by modifying turgor pressure or membrane fluidity, and some are also sensed as mechanical stimuli by living cells. Two classes of putative mechanosensors are currently under investigation, stretch-activated channels and transmembrane proteins inserted into the cell wall/plasma membrane/cytoskeleton (CWPMC) network (Monshausen and Gilroy, 2009; Haswell and Monshausen, 2013).

Two types of stretch-activated channels have been identified so far. Mid1-complementing activity (MCA) proteins are calcium channels (Nakagawa et al., 2007; Yamanaka et al., 2010) and MscS-like (MSL) family members are non-selective channels identified based on homology to bacterial MscS (Kung, 2005; Haswell et al., 2008). Is the rapid desensitization of calcium influx after repeated cold and mechanical sensing linked to the gating kinetics of these channels? In bacteria, results of patch-clamp experiments have indeed shown that MscS channels are inactivated after prolonged exposure to membrane tension (Koprowski and Kubalski, 1998; Levina et al., 1999). In plants, the only gating kinetics obtained for a stretch-activated channel was for the *Arabidopsis* MSL10 channel, but no inactivation of the channel was detected (Maksaev and Haswell, 2012).

The initial sensor of mechanical stress could also be a component of the CWPMC network like receptor-like kinase (RLK) transmembrane proteins (Monshausen and Gilroy, 2009; Haswell and Monshausen, 2013). Whereas a direct link between membrane protein kinases and mechanosensing has not been established, several reports suggest that the cytoskeleton, through its tethering with transmembrane proteins, could be involved. In the *Arabidopsis* meristem, cortical microtubules re-oriented rapidly (within 6 h) in the presence of a mechanical stress (Hamant et al., 2008). A more recent study demonstrated that katanin, a microtubule-severing protein, is required for cell responsiveness to the mechanical stresses generated by growth in *Arabidopsis* meristem cells (Uyttewaal et al., 2012). This provides the outline for a model in which microtubule dynamics allow the cell to respond efficiently to mechanical forces (Nick, 2013).

### NEGATIVE FEEDBACK FROM LONG-TERM ACCUMULATION OF SIGNALING MOLECULES OR TRANSCRIPTION FACTORS

Peaks of cytosolic calcium accumulation are a common feature in many stress signaling pathways. As this calcium response is attenuated by repetitive stimulation, Ca^2+^ influx and eﬄux transporters regulating calcium homeostasis could be considered as components of a “mechanical memory.” Cyclic nucleotide-gated channels (CNGCs) can mediate fluxes of Ca^2+^ ions, and binding of Ca^2+^/calmodulin inactivates CNGCs (Hua et al., 2003; Ali et al., 2006). Thus, the negative action of calmodulin or calmodulin-like proteins on CNGC activity could diminish plant responsiveness through a direct feedback pathway restricting Ca^2+^ influx into plant cells. Genes encoding calmodulin or calmodulin-like proteins are up-regulated early after touch or stem bending (Depège et al., 1997; Lee et al., 2005; Martin et al., 2009), but the involvement of CNGC channels has not been directly addressed in relation to mechanical loading. Glutamate receptors are nonselective cation channels activated by glutamate and glycine (Qi et al., 2006; Stephens et al., 2008) that mediate an increase in cytosolic Ca^2+^ upon cold stress and touch (Meyerhoff et al., 2005). When a second glutamate treatment was applied after the first stimulus, no additional Ca^2+^ response was observed, suggesting that these receptors remain in a putative desensitized state for 1 h (Meyerhoff et al., 2005). Again, the potential role of such glutamate receptors in the mechanotransduction pathway needs to be established.

The desensitization process could also involve transcription factors exerting a negative feedback control on the first stages of the mechanotransduction pathway. In poplar, *PtaZFP2*, a gene encoding a C2H2 transcription factor, is rapidly up-regulated after stem bending (Martin et al., 2009). In *PtaZFP2-*overexpressing poplars, the up-regulation of several mechanoresponsive genes was much weaker after stem bending than in wild-type plants (Martin et al., 2014). Thus, PtaZFP2 negatively modulates poplar responsiveness to mechanical stimulation. Among the genes downstream of PtaZFP2, *CML42*, a calmodulin-like encoding gene, and *WRKY53* and *WRKY40*, transcription factor encoding genes are up-regulated. The *Arabidopsis* homologs of these poplar genes have been described as negative regulators of plant defense responses (Xu et al., 2006; Vadassery et al., 2012). Thus, in concert with these molecular suppressors, PtaZFP2 could reduce the reactivation of the mechanical signaling pathway when stems are bent again.

### INFORMATION STORAGE THROUGH EPIGENETIC REGULATION

One intriguing result is that desensitization of the expression of mechanosensitive genes to mechanical stimuli lasts at least three days (Martin et al., 2010). Bruce et al. (2007) suggested epigenetic changes could be a mechanism for long-term information storage after various abiotic stresses. Chromatin remodeling during drought and cold stresses, which alters accessibility of genes to proteins regulating transcription, has received more attention recently (Kim et al., 2008, 2010; Chinnusamy and Zhu, 2009; To and Kim, 2014). For example, the dynamics of the chromatin status of four stress-responsive candidate genes were analyzed during recovery (i.e., rehydration) of *Arabidopsis* from drought stress (Kim et al., 2012). These studies focused on changes in acetylation (ac) and methylation (me) of lysine (K) residues of histone H3 N-terminal tails. While the proportion of H3K9ac was reduced rapidly during rehydration, H3K4me3 decreased more gradually and was maintained at low levels on the drought-inducible genes even up to 5 h after rehydration. As H3K4me3 is correlated with positive gene responsiveness, the authors suggested that this epigenetic mark of stress memory might help plants respond more effectively to subsequent stresses (Kim et al., 2012; To and Kim, 2014). Indeed, in the *Arabidopsis* H3K4 methyltransferase mutant *atx1*, which is defective in methylating H3K4, the responsiveness of drought-stress inducible genes during the second stress exposure does not increase as much as in wild-type (Ding et al., 2011, 2012).

In an example of cold sensing, the *hos15 Arabidopsis* mutant showed modifications in both histone deacetylation and cold tolerance (Zhu et al., 2008). HOS15 is a WD-40 protein similar to human transducin-beta like protein, a component of repressor complexes involved in histone deacetylation. Apart from an analysis of DNA methylation in *Bryonia* internodes stimulated by rubbing (Galaud et al., 1993), information about epigenetic regulation during plant responses to mechanical loads is scarce. However, some structural features of the aforementioned poplar transcription factor PtaZFP2 indicate that it could be involved in histone modification. The PtaZFP2 protein contains a DLN-box (Gourcilleau et al., 2011), also known as an ERF-associated amphiphilic repression motif (Ohta et al., 2001; Kazan, 2006). Recently, transcription factors containing this motif were reported to exhibit repression activity via histone deacetylation and stimulation of heterochromatin formation in *Arabidopsis* (Causier et al., 2012), so this is preliminary evidence on which to base wider study of epigenetic regulation of responsiveness to mechanical stimuli.

## CONCLUSION

The regulation of plant mechanosensing could be an important part of acclimation to wind by modulating the magnitude and duration of the response and preventing costly investment in reducing or redirecting growth. Modulation of responsiveness could also occur in response to internal mechanical signals produced during tissue or cellular expansion or maturation as part of morphogenesis. The mechanisms underlying this phenomenon are largely unknown essentially because there is so little data on the nature of the presumed mechanosensors or the timing of the regulation. Although some mechanisms are beginning to be identified for other abiotic stresses, they remain largely hypothetical in the case of mechanical stimulation such as bending caused by wind. More high-resolution description of the timing of plant responses at the tissue and cellular level would help to demonstrate the importance of mechanosensing regulation for plant acclimation.

## Conflict of Interest Statement

The authors declare that the research was conducted in the absence of any commercial or financial relationships that could be construed as a potential conflict of interest.
